# Development of the corpus callosum and cognition after neonatal encephalopathy

**DOI:** 10.1002/acn3.51696

**Published:** 2022-12-08

**Authors:** Hollie Byrne, Arthur P. C. Spencer, Georgia Geary, Sally Jary, Marianne Thoresen, Frances M. Cowan, Jonathan C. W. Brooks, Elavazhagan Chakkarapani

**Affiliations:** ^1^ Clinical Research and Imaging Centre University of Bristol Bristol UK; ^2^ Murdoch Children's Research Institute Melbourne Victoria Australia; ^3^ Department of Paediatrics University of Melbourne Melbourne Victoria Australia; ^4^ Translational Health Sciences, Bristol Medical School University of Bristol Bristol UK; ^5^ Royal Glamorgan Hospital Cwm Taf University Health Board Abercynon UK; ^6^ Faculty of Medicine, Institute of Basic Medical Sciences University of Oslo Oslo Norway; ^7^ Department of Paediatrics Imperial College London London UK; ^8^ University of East Anglia Wellcome Wolfson Brain Imaging Centre (UWWBIC) University of East Anglia Norwich UK; ^9^ Neonatal Intensive Care Unit, St Michaels Hospital University Hospitals Bristol and Weston NHS Foundation Trust Bristol UK

## Abstract

**Objective:**

Neonatal imaging studies report corpus callosum abnormalities after neonatal hypoxic–ischaemic encephalopathy (HIE), but corpus callosum development and relation to cognition in childhood are unknown. Using magnetic resonance imaging (MRI), we examined the relationship between corpus callosum size, microstructure and cognitive and motor outcomes at early school‐age children cooled for HIE (cases) without cerebral palsy compared to healthy, matched controls. A secondary aim was to examine the impact of HIE‐related neonatal brain injury on corpus callosum size, microstructure and growth.

**Methods:**

Participants aged 6–8 years underwent MRI, the Movement Assessment Battery for Children Second Edition and Wechsler Intelligence Scale for Children Fourth Edition. Cross‐sectional area, volume, fractional anisotropy and radial diffusivity of the corpus callosum and five subdivisions were measured. Multivariable regression was used to assess associations between total motor score, full‐scale IQ (FSIQ) and imaging metrics.

**Results:**

Adjusting for age, sex and intracranial volume, cases (*N* = 40) compared to controls (*N* = 39) demonstrated reduced whole corpus callosum area (*β* = −26.9, 95% confidence interval [CI] = −53.17, −0.58), volume (*β* = −138.5, 95% CI = −267.54, −9.56), fractional anisotropy and increased radial diffusivity (*P* < 0.05) within segments II–V. In cases, segment V area (*β* = 0.18, 95% CI = 0.004, 0.35), volume (*β* = 0.04, 95% CI = 0.001, 0.079), whole corpus callosum fractional anisotropy (*β* = 13.8 95% CI = 0.6, 27.1) and radial diffusivity (*β* = −11.3, 95% CI = −22.22, −0.42) were associated with FSIQ. Growth of the corpus callosum was restricted in cases with a FSIQ ≤85, and volume was reduced in cases with mild neonatal multifocal injury compared to white matter injury alone.

**Interpretation:**

Following neonatal HIE, morphological and microstructural changes in the corpus callosum are associated with reduced cognitive function at early school age.

## Introduction

Therapeutic hypothermia (TH), reducing the body temperature to 33.5°C for 72 h, has reduced the incidence^1^, ^2^ and severity^3^ of cerebral palsy (CP) in infants with neonatal hypoxic–ischaemic encephalopathy (HIE) following perinatal asphyxia. However, cooled children who do not develop CP have demonstrated white matter (WM) connectivity perturbations,^4^ and cognitive and motor impairments relative to their healthy peers at early school age.^5^ Previous neonatal magnetic resonance imaging (MRI) studies in cooled infants identified abnormal corpus callosum (CC) signal^6^ and microstructural integrity,^7^ which were associated with death or neurodevelopmental delay and cognitive performance before 2 years of age. However, neonatal CC diffusion abnormalities can be influenced by TH,^8^ injury to other brain regions^6^ and oedema that follows an acute injury.^9^ Additionally, as the CC matures beyond the neonatal period^10^ and cognitive skills are less robustly assessed before school age,^11^ later imaging is needed to observe the altered developmental trajectory of the CC and associated outcomes. While CC abnormalities have been described in children who develop CP following HIE, no such data exist for children without CP who were cooled for HIE.

To address these issues, we compared CC morphometry and microstructure derived from structural and diffusion‐weighted magnetic resonance imaging (MRI) at early school age (6–8 years) between children without CP cooled for HIE (cases) and matched controls. We then examined the impact of neonatal hypoxic–ischaemic brain injury on CC morphology at early school age, and the association between cognitive and motor impairment at early school age and CC growth. Finally, we investigated the association between CC morphology, microstructure and cognitive and motor function at early school age in cases and controls.

## Methods

### Standard protocol approvals, registrations and patient consent

The South West‐Frenchay NRES Committee and Health Research Authority, UK (15/SW/0148) approved the study. We recruited a prospective cohort after obtaining informed parental consent. The study was conducted at the University of Bristol, UK.

### Participants

#### Cases

Sixty‐nine eligible case children were identified from a patient database at St Michael's Hospital, Bristol, UK. Of those contactable, 50 agreed to participate. We included children aged 6–8 years, born >35 weeks' gestation at St Michael's Hospital who underwent TH within 6 h of birth for encephalopathy, confirmed by neurological examination and amplitude‐integrated electroencephalogram (aEEG),^12^, ^13^ following perinatal asphyxia. Children cooled outside standard criteria, who developed CP, had additional medical/psychological diagnoses or did not have English as their primary spoken language were excluded. CP was ruled out at 2 and 6–8 years by a consultant paediatrician or an experienced physiotherapist using a standard neurological assessment of motor function, muscle tone and deep tendon reflexes.

#### Controls

Children aged 6–8 years, born >35 weeks' gestation without neonatal HIE were recruited via local schools in Bristol. Of the 65 parents who registered interest in the study, we recruited 43 eligible children most comparable to cases. Cases and controls were matched at the group level by age, sex and socio‐economic status as determined by the index of multiple deprivations, based on a weighted combination of seven domains, as defined by the UK Government.^14^ The control group had no contraindications to MRI or pre‐existing medical/psychological diagnoses, and their primary spoken language was English.^5^

### MRI data acquisition

#### Neonatal (cases only)

Neonatal MRI scans were performed using a 1.5T Siemens Symphony (Erlangen, Germany) between 4 and 15 days after birth, during natural sleep following a feed or with chloral hydrate if required. All infants underwent T1‐weighted (T1‐W) and T2‐W imaging using turbo spin echo sequences (slice thickness 4 mm), echo time (TE)/repetition time (TR) = 7.7/400 ms, flip angle 90° and TE/TR = 99/3520 msec. Axial diffusion‐weighted imaging (DWI) was obtained using TE/TR: 112/3700 msec, flip angle 90° and three diffusion weightings (*b* = 0, 500, 1000 s/mm^2^). T1‐W, DWI and T2‐W scans were used for qualitative inspection of the CC and sagittal T1‐W images were used for manual morphometric CC analysis.

#### Childhood

Participants underwent MRI using a 3T Siemens Skyra. The protocol included a T1‐W sequence (magnetisation prepared rapid gradient echo): 176 slices, 1.0 × 1.0 × 1.0 mm voxels, TE/TR = 2.19/1500 msec, inversion time = 800 msec, flip angle 9°, field of view (FoV) 234 × 250 mm, GeneRalized Autocalibrating Partially Parallel Acquisitions (GRAPPA) acceleration factor 4^15^; T2 turbo spin echo: TE/TR = 11/10130 msec, thickness 4.0 mm; DWI: 60 slices, TE/TR = 70/3500 msec; flip angle 90°, FoV 192 × 192 mm, 2.0 × 2.0 × 2.0 mm voxels, multi‐band acceleration factor 2, GRAPPA acceleration factor 2 and echo planar imaging.^16^, ^17^ Images were acquired with *b* = 1000 sec/mm^2^ in 60 diffusion directions, equally distributed according to an electrostatic repulsion model, as well as 8 interspersed *b* = 0 sec/mm^2^ images. For eddy current and distortion correction, two sets of diffusion data were acquired with opposite phase encoding polarity (AP/PA directions).

Before scanning, children were shown a video demonstrating the MRI procedure and chose a DVD to watch during scanning. Scans were individually inspected by two raters (J. C. Brooks and A. P. Spencer) blinded to case–control status for suitability for analysis, excluding scans with significant motion artefacts.

### Qualitative assessment of structural imaging

The presence and extent of brain injury on neonatal and childhood MRI was assessed by a perinatal neurologist experienced in MRI interpretation (F. M. Cowan) blinded to case–control status and independently of the neonatal scans. Neonatal brain injury was quantified using the Rutherford classification system,^18^ a robust predictor of developmental outcome following neonatal HIE.^19^ Using this system, the basal ganglia and thalami (BGT), cortex and WM are each scored from 0 to 3 and the posterior limb of the internal capsule (score 0–2) and summed to give a total injury score (TIS: 0 [no injury] to 11 [maximum injury]).^18^, ^19^ As the case/cohort was limited to children without CP, neonatal brain injury scores were low. Therefore, we classified regional brain injury scores into no BGT injury (BGT score 0), BGT injury (BGT score 1–3), no WM injury (WM score 0) or WM injury (WM score 1–3). To assess the impact of BGT and WM injury on childhood CC morphometry, we classified four injury severity groups: (1) no injury, (2) BGT injury only, (3) WM injury only and (4) combined BGT and WM injury.

Childhood MRI scans were assessed for the presence of abnormalities, particularly focal central grey matter (GM) lesions and WM hyperintensities on T2‐W images.

### Childhood CC topography

As CC size and thickness depend on axonal fibre density,^20^ we measured CC area, volume and microstructure. To reduce bias, automated and manual measurements were conducted by assessors blinded to case status. The CC was divided into five segments based on the Hofer and Frahm topographical atlas,^21^ where segments reflect projections to different cortical territories. These include projections to the prefrontal (segment I), premotor and supplementary motor (segment II), primary motor (segment III), primary sensory regions (segment IV) and to parietal, temporal and occipital areas (segment V).

### CC area and volume

#### Manual CC parcellation

##### Neonatal

Thirty‐six (85.7%) cases with childhood MRI had neonatal imaging suitable for morphometric analysis. Manual calculations were undertaken by a single assessor (G. Geary). Longitudinal total and anterior‐, mid‐ and posterior‐third cross‐sectional CC area and supratentorial brain area (STBA) were calculated from mid‐sagittal T1‐W images by manually demarcating the boundaries of the CC and brain using Osirix.^22^

The process was repeated three times and averaged. As the Hofer and Frahm classification has not been validated in neonatal cohorts, we used anterior, middle and posterior CC subdivisions commonly reported with manual segmentation. We further subdivided the middle‐third into the anterior‐third and posterior two‐thirds as this latter section carries most motor fibres. We computed the ratio of whole and CC subdivisions area to the STBA to adjust for differences in brain size.

##### Childhood

Manual calculations were performed by the same assessor (G. Geary) blinded to case–control status, using the method described above.

#### Automated CC parcellation of childhood scans

##### Area

Morphometric analysis was conducted using ‘C8’, a fully automated MATLAB program for quantifying the area of the human CC.^23^ First, T1‐W images were brain extracted using VBM8 (part of SPM8)^24^ and segmented into central and cortical GM, WM and cerebrospinal fluid maps using FAST (part of FSL).^25^, ^26^, ^27^ T1‐W scans and segmented WM images were then registered to an age‐appropriate MNI template from the Neurodevelopmental MRI Database (http://jerlab.psych.sc.edu/NeurodevelopmentalMRIDatabase/) using rigid body transformation with FSL's FLIRT.^27^, ^28^ Registered brain and WM maps were input to C8, which applied a probability threshold of 0.9 to identify and measure the CC. C8 calculates the callosal area from the mid‐sagittal plane (*x* = 0) and four adjacent parasagittal slices (*x* = −2, −1, 1, 2) to provide a median value for the area for each subject. The area of each of the five segments of the CC according to Hofer and Frahm was calculated.^21^

##### Volume

Callosal volume estimates were generated by summating the areas calculated for the five slices (each 1 mm thick) spanning the midline. Corresponding volumes for each of the five Hofer and Frahm segments were estimated by multiplying the whole CC volume by the relative proportions for each of the segment areas.

### Intracranial volume

Intracranial volumes (ICVs, including cerebellum) were calculated for childhood scans using partial volume estimate (PVE) segmentations obtained from FAST. Using the brain‐extracted images, the mean voxel PVE for GM, WM and cerebrospinal fluid across the whole image for each subject were multiplied by the total image volume (mm^3^), ignoring all voxels with a value of zero. The total volume of each tissue type was summed to produce ICV. Due to movement and poor alignment between scans, ICV could not be calculated for the neonatal data set.

### CC microstructure

Diffusion‐weighted imaging data were corrected for bias, motion and distortion using the Eddy and Topup tools from FSL.^25^, ^29^, ^30^ The diffusion tensor model was fitted to diffusion images using the weighted least squares method in FSL's FDT software, generating fractional anisotropy (FA) and radial diffusivity (RD) images. Voxelwise statistical analyses of FA and RD data were carried out using FSL's tract‐based spatial statistics (TBSS).^31^ Following recommendations for studies involving children,^31^ all FA images were non‐linearly registered to a study‐specific target image, which was selected from the study cohort to minimise total deformation required to perform all registrations, then linearly registered to MNI152 standard space. The mean FA image was thresholded at 0.2 to create a skeletonised representation of the WM tracts. A mask was generated for the CC using the JHU ICBM‐81 atlas,^32^ which was proportionately divided into the five Hofer and Frahm segments.^21^ This mask was then used to perform a region of interest analysis on the FA skeleton for cases versus controls. Within each region of interest, a voxelwise assessment of FA was performed as described below. For each participant, transformations derived from the FA analysis were also applied to RD images. We also obtained mean FA and RD within the WM skeleton in each CC segment and the whole CC for each participant.

### Psychometric and motor assessments

Psychologists blinded to case–control status assessed cognitive performance (full‐scale IQ, FSIQ) using the Wechsler Intelligence Scale for Children 4th Edition (WISC‐IV).^33^ Motor performance was assessed by blinded researchers using the Movement Assessment Battery for Children Second Edition (MABC‐2),^34^ as described in previous work.^5^ Cognitive and motor impairment were defined as FSIQ ≤85 and MABC‐2 total score ≤15th centile respectively.

### Statistical analysis

Normality distribution for demographics in the neonatal and childhood period was assessed using the Shapiro–Wilk's test. Independent samples *t* test was used to compare the normally distributed variables and Mann–Whitney *U* to compare variables of skewed distribution. Proportions were compared using chi‐squared tests. To reduce bias and improve reliability, the relationship and agreement between childhood manual and automated whole CC area measurements was assessed using a scatter plot, Pearson correlation coefficient and Bland–Altman plot.

#### Case–control comparison of childhood CC metrics

The relationship between case status and whole/individual CC segment areas and volumes derived from C8 was analysed using univariable linear regression and adjusted for age at scan, sex and ICV using multivariable linear regression. Using stepwise multivariable linear regression, the effect of sex on whole or individual CC segment areas and volumes/ICV ratio was explored with case–control status, age at scan and the interaction variable ‘case status*sex’ as covariates. For microstructural analysis with TBSS, a voxelwise comparison of diffusion metrics and association with cognitive and motor scores was performed using FSL's non‐parametric permutation testing software, RANDOMISE.^35^ We tested for case–control differences in FA and RD, and for ‘case–control status*sex’ interaction. We then tested for ‘case–control status*FSIQ’ interaction and ‘case–control status*MABC‐2 total score’ interaction with both FA and RD. Age and sex were included in a general linear model in all tests. We used 10,000 permutations and applied threshold‐free cluster enhancement^36^ to correct for multiple comparisons. Significant results have a family‐wise error rate *P*< 0.05.

#### Relationship between neonatal brain injury and childhood CC metrics (cases only)

Association between TIS assessed from neonatal MRI and childhood CC area and volume normalised to ICV was assessed with simple linear regression. The Kruskal–Wallis test was used to compare childhood whole CC area and volume normalised to ICV, RD and FA between controls and (1) cases with TIS score 0–3 or TIS score 4–6, and (2) cases with no neonatal BGT/WM injury, BGT injury only, WM injury only and both BGT and WM injury. Post‐hoc tests were corrected for multiple hypothesis testing using Benjamini‐Hochberg. A false discovery rate (FDR) ≤5% was considered significant.^37^

FSIQ, MABC‐2 total score, WISC‐IV and MABC‐2 domain scores were compared between cases and controls using independent samples t tests and multiple comparisons correction using FDR (as above). Manually parcellated areas of the whole and three CC subdivisions on neonatal and childhood scans were used to assess CC growth. Multivariate regression analyses for repeated measures (neonatal and childhood whole CC and CC subdivision areas) using generalised estimating equations were applied using Gaussian family distribution, linked by participant identification number, and independent correlation structure with robust standard error adjustment to analyse whether the growth of the whole CC and CC subdivision areas were related to FSIQ≤ or >85 or MABC‐2 ≤ or >15th centile controlled for age at scan as a covariate. Stepwise multivariable linear regression was used to determine associations between childhood whole CC and segments area, volume, FA, RD and outcome variables FSIQ and MABC‐2 total score adjusted for covariates sex, age at scan, case status and ICV. This was computed for cases and controls together and separately.

All analyses were conducted using IBM SPSS Statistics for Windows, Version 27.0 (Armonk, NY) and graphs were produced in GraphPad Prism 8.0 for Windows (GraphPad Software, La Jolla, CA, https://www.graphpad.com/scientific‐software/prism/). All *P*‐values reported are two‐tailed and <0.05 is considered significant.

## Results

**Figure 1:**
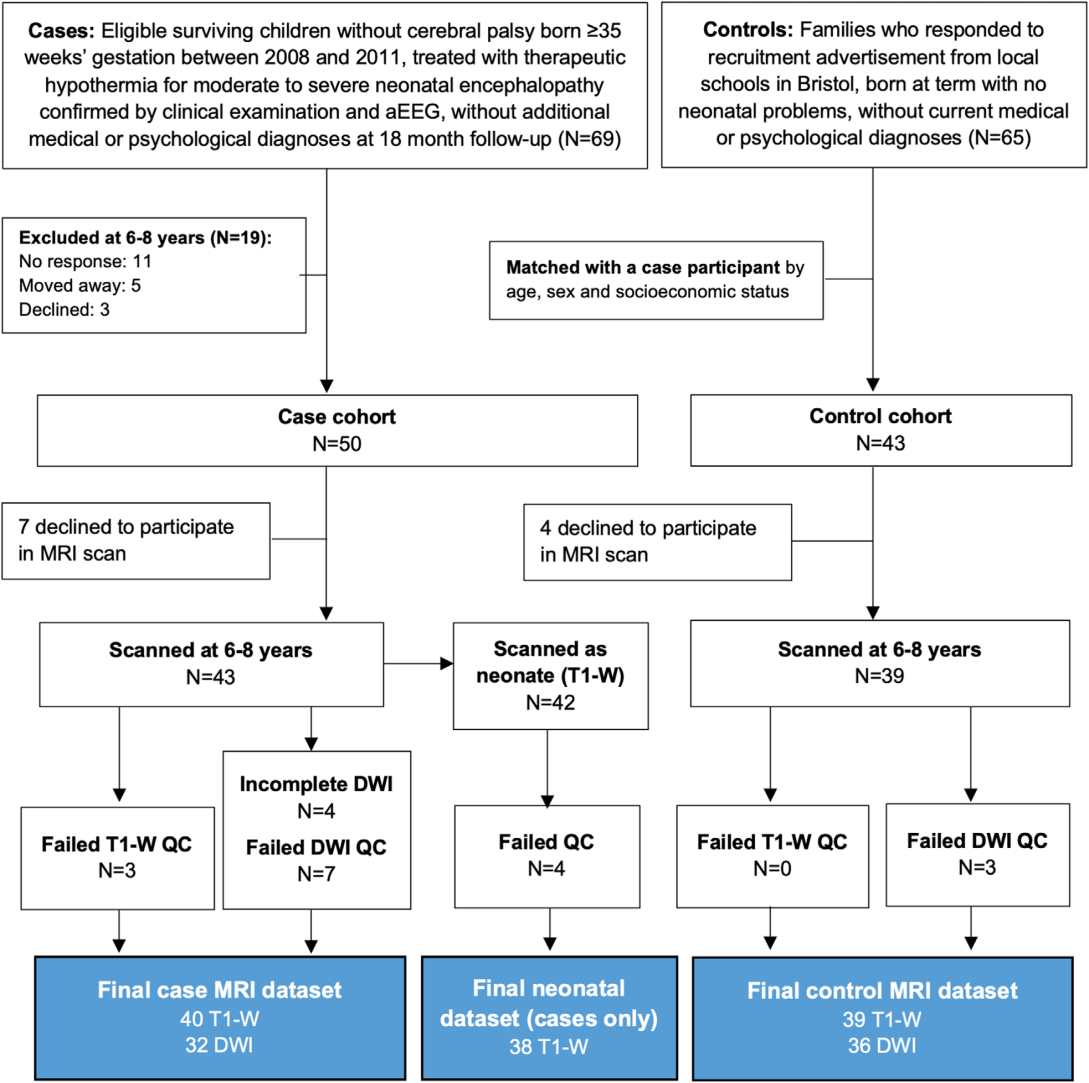
Recruitment diagram.

Of 93 participants recruited, 11 (7 cases) did not want to participate in the MRI scan. Of the 82 scans obtained, 3 cases (0 controls) failed T1‐W quality control, leaving 79 participants (40 cases) with suitable quality T1‐W data for morphometric analysis. Of the 82 DWI scans acquired, 4 cases had incomplete data, and 7 cases and 3 controls failed DWI quality control, leaving 68 participants (32 cases) for analysis (Fig. 1). More details of quality control and factors associated with rejected scans in this cohort is presented in Woodward et al.^38^ Demographic and neonatal characteristics are given in Table 1. Cases had a median (interquartile range) 10‐min Apgar score of 6 (5, 8), and pH and base excess within 1 h of birth of 6.95 (6.82, 7.10) and −16.2 (−19.9, −13.0). Most cases (95%) had a moderately abnormal and 5% severely abnormal aEEG pattern before TH.

**Table 1:** Comparison of baseline characteristics between cases and controls who were scanned at 6–8 years.

Variable	_Anatomical analysis_			_DWI analysis_		
	Cases (*N* = 40)	Controls (*N* = 39)	*P* value	Cases (*N* = 32)	Controls (*N* = 36)	*P* value
Neonatal characteristics						
Birthweight (g), mean (SD)	3400 (536)	3549 (488)	0.202	3386 (559)	3501 (452)	0.351
Gestation at birth, mean (weeks), (SD)	39.8 (1.3)	40.1 (1.4)	0.313	39.9 (1.4)	40.1 (1.4)	0.519
Male sex, *N* (%)	21 (52.5)	22 (56.4)	0.727	16 (50.0)	21 (58.3)	0.491
Characteristics at 6–8 years						
Age at testing in months, mean (SD)	84.4 (6.3)	83.5 (6.6)	0.542	83.7 (6.0)	83.6 (6.7)	0.959
Height in cm, mean (SD)	124 (6.2)	124 (6.0)	0.537	123 (6.4)	124 (6.0)	0.571
Weight in kg, mean (SD)	26.2 (5.2)	24.7 (3.9)	0.164	25.7 (5.0)	24.5 (3.9)	0.276
Head circumference in cm, mean (SD)	52.7 (2.2)	52.6 (1.4)	0.924	52.3 (2.2)	52.6 (1.5)	0.567
Multiple deprivation scores, median (IQR)	7 (5)	7 (3.5)	0.385	6.5 (5)	7 (4)	0.403

IQR, interquartile range; SD, standard deviation.

### Qualitative MRI assessment

#### Neonatal

Scans (*N* = 42) were obtained at a mean (standard deviation [SD]) age of 7.9 (1.97) days; 12/42 (28.6%) showed no evidence of brain injury (TIS = 0). BGT scores of 2 and 1 were ascribed to 2 and 6 scans respectively (8/42 = 19%) and WM scores of 3, 2 and 1 were ascribed to 3, 11 and 16 scans respectively (30/42 = 71.4%). No CC signal abnormalities were seen on any image. All eight scans with evidence of BGT injury also showed WM injury (BGT score 2, WM score 1 [*N* = 2]; BGT score 1, WM score 2 [*N* = 3] and WM score 1 [*N* = 3]). The remaining 22 scans with WM injury (3 score 3, 8 score 2, 11 score 1) had normal BGT.

#### Childhood

One case (neonatal TIS score 2) and one control had a small focal lesion with an increased T2 signal in the right trigone.

### Manual versus automated CC area comparison at childhood

In 79 participants (40 cases), manual and automated whole CC area measurements were correlated (*r* ^2^ = 0.87, *P* = 0.0001) with manual measurements smaller on average: mean difference (SD) 32.64 mm^2^ (39.62). The limits of agreement were −1110.3 to 45.01 with three data points (3.6%) outside. To reduce human error, we used automated CC area and volume measurements for all analyses except CC growth, as data from automated analysis of neonatal CC area were unavailable.

### Differences in childhood CC morphology and microstructure between cases and controls

#### Morphometrics

Cases had lower ICV (mean difference [95% CI]: 77.1 cm^3^ [19.8, 134.4]), whole CC area (*β* [95% CI]; −49.3 [−78.60, −20.01]) and volume (−250.7 [−395.51, −106.0]), area of CC segments I (−10.6 [−19.87, −1.45]), II (−17.6 [−28.25, −6.94]), III (−4.3 [−8.1, −0.48]) and V (−14.6 [−25.65, −3.56]) and volume of CC segments I (−56.2 [−99.28, −13.16]), II (−85.5 [−137.46, −35.61]), III (−22.2 [−40.2, −4.23]) and V (−74.4 [−126.03, −22.72]). When adjusting for ICV, sex and age at scan, cases demonstrated reduced CC segment II area (−11.2 [−21.28, −1.13]) and volume (−54.22 [−101.71, −6.72]), and whole CC area (−26.9 [−53.17, −0.58]) and volume (−138.5 [−267.54, −9.56]) compared to controls.

#### Effect of sex on morphometrics

In cases, males had higher ICV than females (mean difference [95% CI] 138.8 cm^3^ [65.8, 211.9]); there was no sex difference in controls (71.0 cm^3^ [−6.9, 148.9]). In the multivariable regression, all males compared to all females had lower CC segment III area/ICV ratio (*β* [95% CI]; −4.0 × 10^−6^, [−7.0 × 10^−6^, −1.0 × 10^−6^]), segment III volume/ICV ratio (−1.6 × 10^−5^, [−2.8 × 10^−5^, −0.5 × 10^−5^]) and whole CC area/ICV ratio (−2.0 × 10^−5^, [−3.8 × 10^−5^, −0.2 × 10^−5^]) independent of the age of scan and case status without interaction with case status. However, male cases compared to female cases and controls had lower whole CC volume/ICV ratio (−11.1 × 10^−5^, [−21.2 × 10^−5^, −0.9 × 10^−5^]) showing an interaction between case status and sex.

#### Microstructure

**Figure 2:**
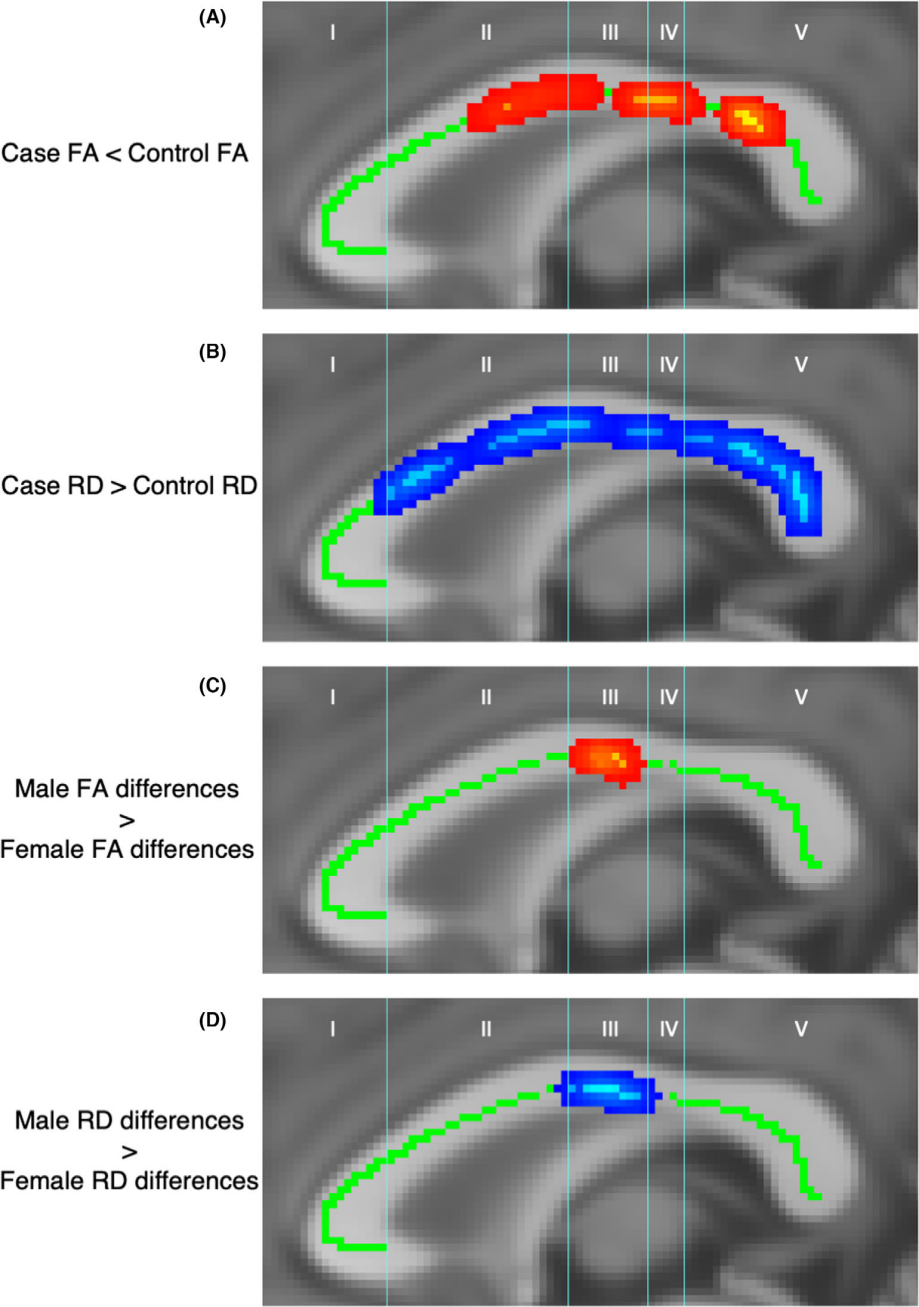
Voxelwise comparison of microstructure on the white matter skeleton (green), using tract‐based spatial statistics, revealed areas of reduced FA in cases (A), areas of increased RD in cases (B), and areas in which males exhibit larger case–control differences in FA (C) and RD (D) than females. Significant differences are indicated by areas of red‐yellow for FA images and blue for RD images (*P* < 0.05). FA, fractional anisotropy; RD, radial diffusivity.

TBSS analysis revealed decreased FA (*P* < 0.05) in CC segments II, III, IV and V in cases compared to controls (Fig. 2A). Cases had increased RD (*P* < 0.05) in the same segments (II, III, IV and V) compared to controls (Fig. 2B). When exploring sex effect, males showed larger case–control differences in both FA (Fig. 2C) and RD (Fig. 2D) in segment III than females.

### Impact of neonatal brain injury on early school‐age CC morphometrics and microstructure

**Figure 3:**
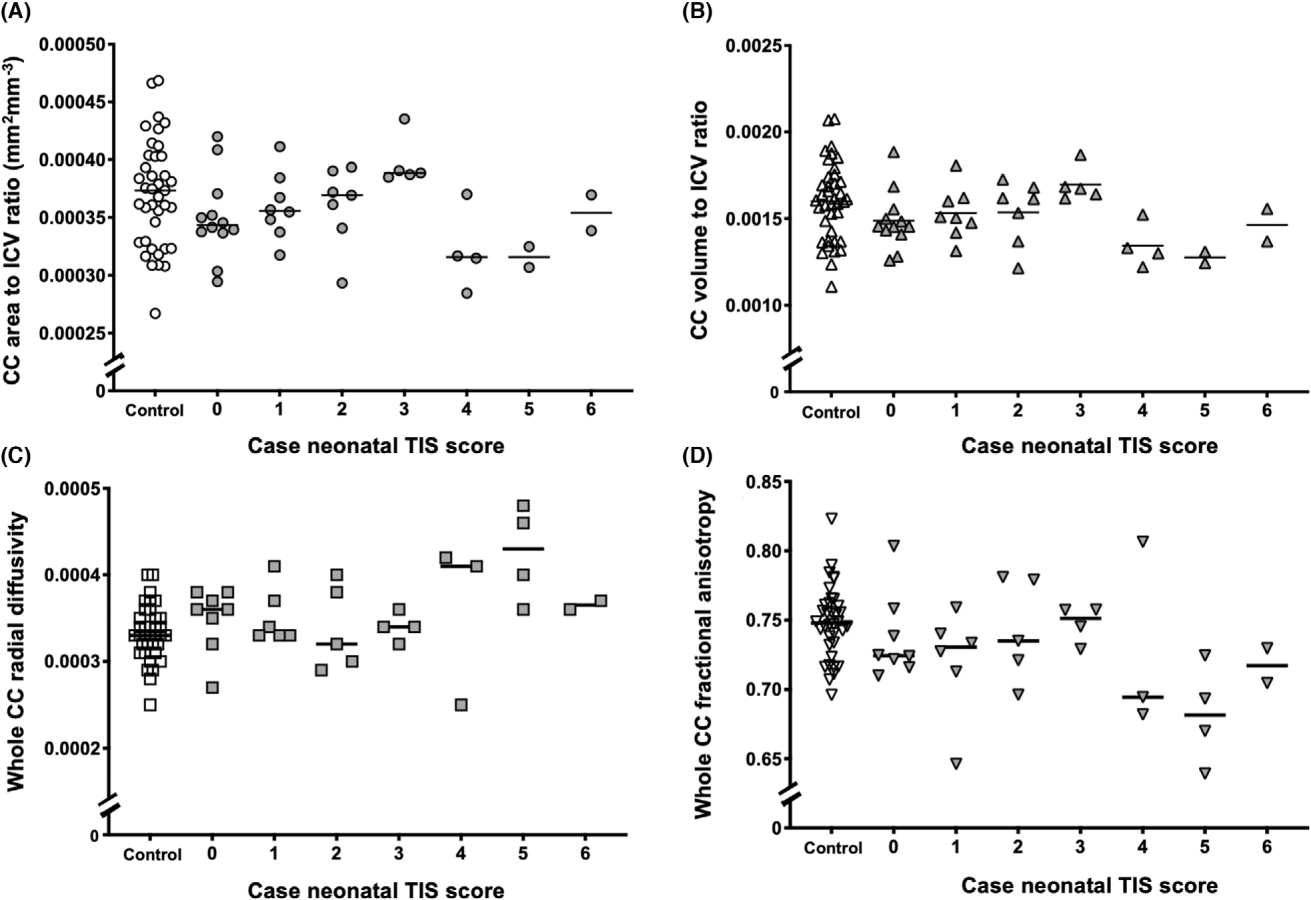
Relationship between CC metrics and neonatal TIS of cases compared to controls. Figures depict CC area and TIS of cases (grey) compared to controls (white) (A); CC volume and TIS of cases compared to controls (B); CC RD and TIS of cases compared to controls (C); CC FA and TIS of cases compared to controls (D). CC, corpus callosum; FA, fractional anisotropy; RD, radial diffusivity; TIS, total injury score.

**Figure 4:**
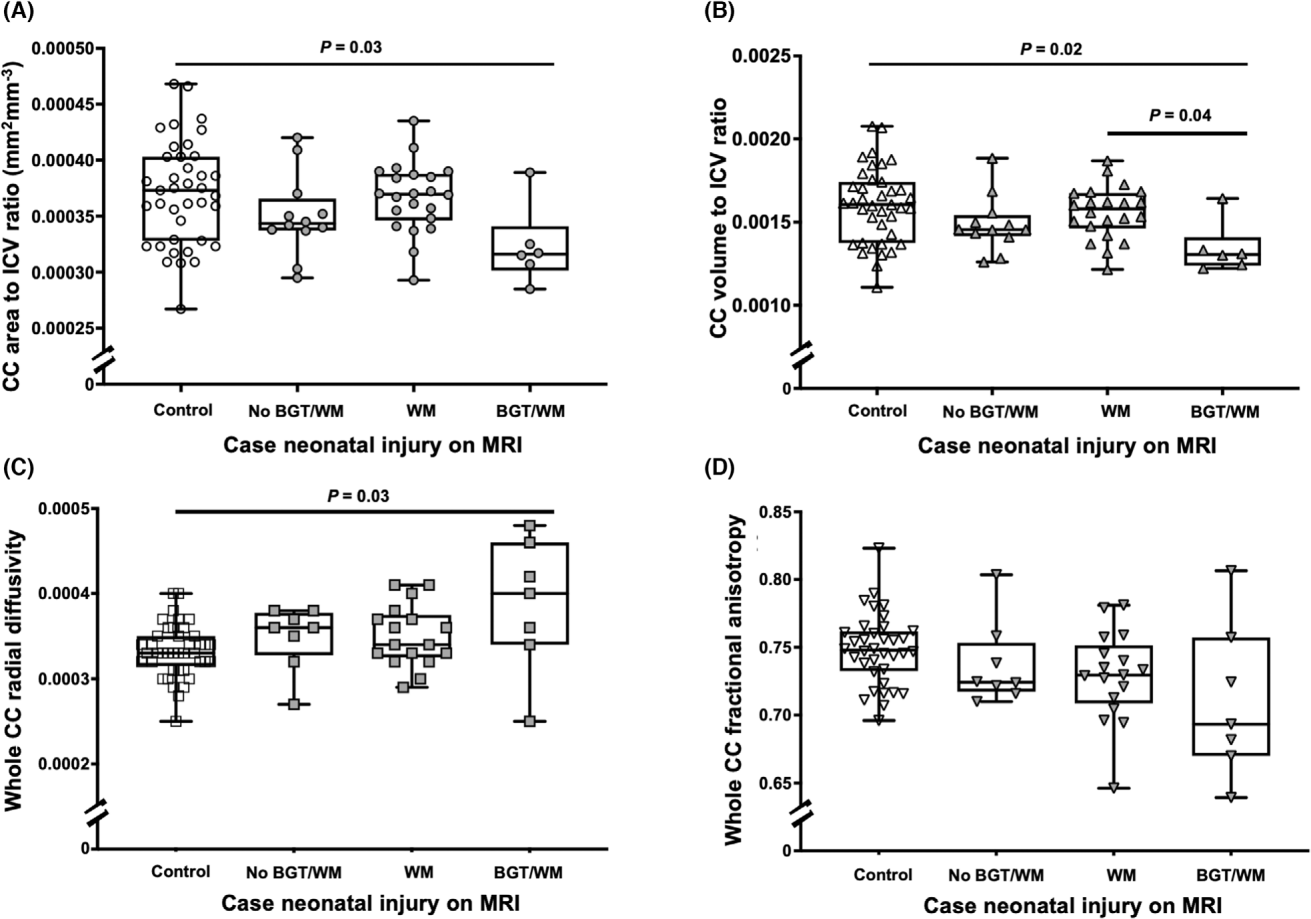
Relationship between CC metrics and presence of neonatal injury in cases compared to controls as identified through simple linear regression. CC area of cases with no BGT/WM, WM only or BGT/WM injury compared to controls (A); CC volume of cases with no BGT/WM, WM only or BGT/WM injury compared to controls (B); CC RD of cases with no BGT/WM, WM only or BGT/WM injury compared to controls (C); CC FA of cases with no BGT/WM, WM only or BGT/WM injury compared to controls (D). Cases no BGT/WM = case with no BGT/WM injury on neonatal MR brain (BGT score 0). Case WM = cases with any WM lesions on neonatal MR brain (WM score 1–3). Case BGT/WM = cases with any BGT lesions on neonatal MR brain (BGT score 1–2 and WM score 1–3). BGT, basal ganglia and thalami; CC, corpus callosum; FA, fractional anisotropy; RD, radial diffusivity; TIS, total injury score; WM, white matter

Corpus callosum area and volume were expressed as a proportion of ICV to account for case–control differences. Of the 42 children with neonatal scans, 40 had morphometric data and 32 had diffusion measurements at early school age. There was no significant linear association between neonatal MRI TIS and whole CC area, volume, RD and FA on early school‐age scans (Fig. 3). However, compared to controls, children with TIS 4–6 had reduced whole CC area (ranks, 44.0 vs. 20.5, *P* = 0.02), volume (ranks, 45.3 vs. 16.8, *P* = 0.004), FA (ranks, 40.4 vs. 16.1, *P* = 0.002) and higher RD (28.9 vs. 52.5, *P* = 0.003). Compared to children with TIS 0–3, children with TIS 4–6 had lower whole CC area (40.0 vs. 20.5, *P* = 0.047) and volume (39.4 vs. 16.8, *P* = 0.02). Controls, cases with no BGT or WM injury and cases with isolated WM injury differed to cases with BGT and WM injury in the whole CC area, volume and RD (Fig. 4). There was no difference in whole CC FA based on neonatal brain injury (Fig. 4D). On post‐hoc testing with multiple comparisons correction, whole CC volume was reduced in cases with BGT and WM injury compared to cases with WM injury only (ranks, 15.8 vs. 41.9, *P* = 0.04) or controls (ranks, 15.8 vs. 45.3, *P* = 0.04).

### Childhood cognitive and motor performance

On average, cases had lower FSIQ and domain scores compared to controls on the Verbal Comprehension, Perceptual Reasoning and Working Memory WISC‐IV sub‐tests (Table 2). A higher proportion of cases demonstrated cognitive and motor impairments and lower WISC‐IV domain scores than controls (Table 2).

**Table 2:** Cognitive and motor performance of cases compared to controls.

Assessment result	Cases (*N* = 40)	Controls (*N* = 39)	Difference (95% CI)	*P* value
FSIQ, mean (SD)	96.4 (12.3)	108 (13.0)	−11.6 (−17.3 to −6.0)	<0.001**
MABC‐2 total score, mean (SD)	9.8 (4.1)	10.7 (2.9)	−0.9 (−2.5 to 0.8)	0.284
WISC‐IV subscales				
Verbal comprehension, mean (SD)	99.4 (11.4)	107.6 (10.1)	−8.1 (−13.0 to −3.3)	0.001**
Perceptual reasoning, mean (SD)	93.3 (12.7)	107.8 (14.1)	−14.5 (−20.5 to −8.5)	<0.001**
Working memory, mean (SD)	95.2 (12.8)	103.9 (12.4)	−8.7 (−14.3 to −3.1)	0.003**
Processing speed, mean (SD)	100.5 (15.8)	105.4 (15.5)	−4.9 (−11.9 to 2.1)	0.168
FSIQ <1 SD below test mean, *n* (%)	7 (17.5)	1 (2.6)		0.020*
FSIQ <2 SD below test mean, n (%)	1 (2.5)	0 (0)		0.338
MABC‐2 subscales				
Aiming and catching, mean (SD)	9.4 (3.6)	10.0 (2.8)	−0.5 (−2.0 to 0.9)	0.450
Balance, mean (SD)	10.4 (4.0)	11.1 (2.8)	−0.7 (−2.3 to 0.9)	0.375
Manual dexterity, mean (SD)	9.7 (3.9)	10.8 (3.1)	−1.1 (−2.7 to 0.5)	0.184
MABC‐2 total score ≤15th centile, *n* (%)	12 (30)	2 (5.1)		0.003**
MABC‐2 total score ≤5th centile, *n* (%)	7(17.5)	1 (2.6)		0.020*

CI, confidence interval; FSIQ, full‐scale IQ; MABC‐2, Movement Assessment Battery for Children – Second Edition; SD, standard deviation; WISC‐IV, Wechsler Intelligence Scale for Children – Fourth edition. Multiple hypothesis testing was addressed using the Benjamini‐Hochberg method with a false discovery rate of ≤5%.

* *P* < 0.05.

** *P* < 0.005.

### Growth trajectory of the CC and behavioural results in cases

**Figure 5:**
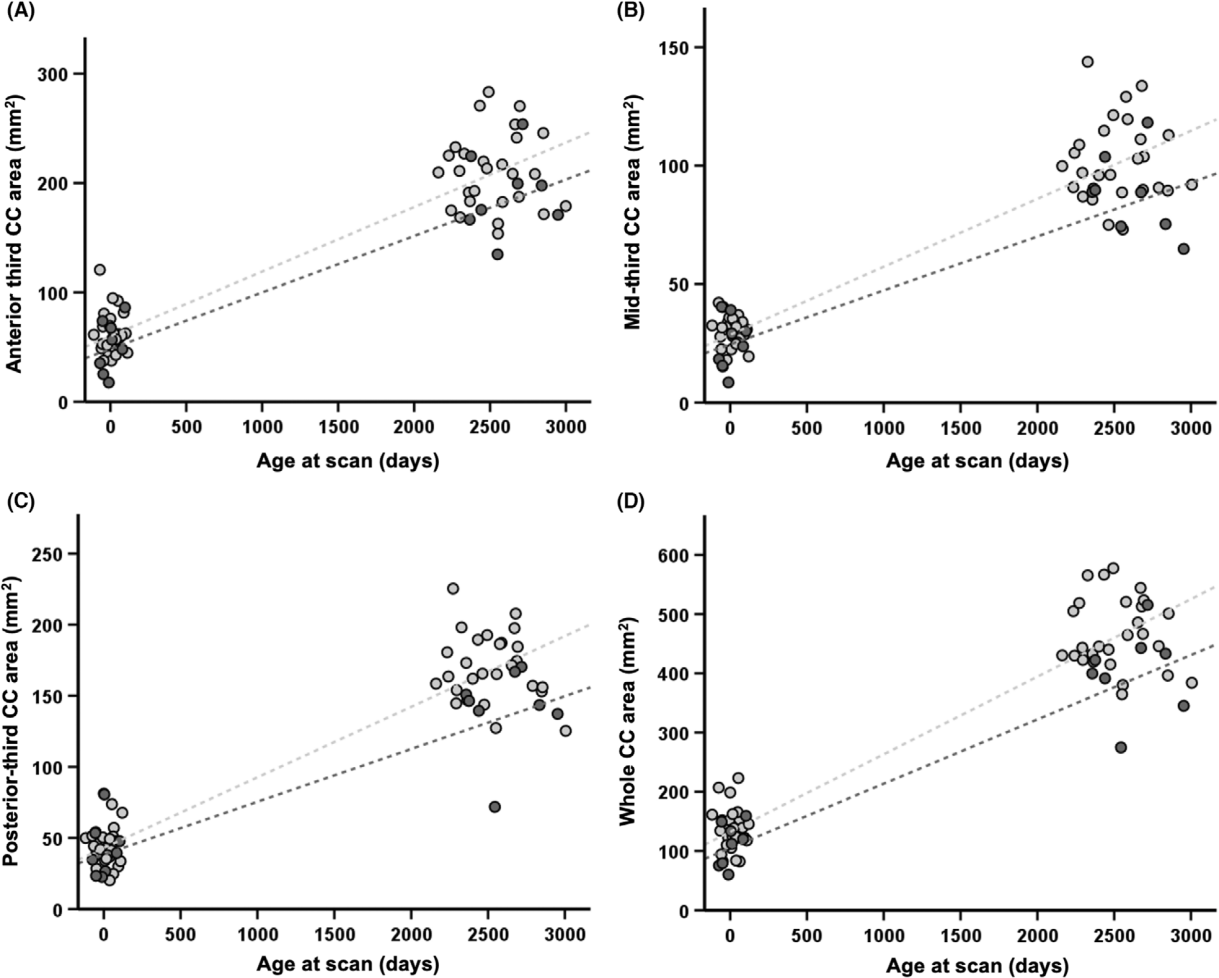
Evolution of anterior (A), mid (B) and posterior (C) third and whole (D) CC area from neonatal to early school‐age MRI (*n* = 36) in cases classified into two groups with an FSIQ ≤85 or >85. Light grey circles represent FSIQ >85, and dark grey circles represent FSIQ ≤85 group. Light and dark grey interrupted lines represent the linear regression lines of the FSIQ >85 and FSIQ ≤85 group. CC, corpus callosum; FSIQ, full‐scale IQ; MRI, magnetic resonance imaging.

Corpus callosum growth trajectory was assessed using the manual CC parcellation measurements from the neonatal and early school‐age scans (*N* = 36). Cases with an FSIQ ≤85 compared to those >85 had a lower slope of anterior, mid, posterior third and whole CC area growth (Fig. 5) independent of age at scan. No association between motor impairment and CC growth was identified.

### Relationship between early school‐age CC morphology and microstructure with motor and cognitive performance

#### Regression

The area and volume of CC segment V, whole CC average FA and average RD were associated with FSIQ independent of ICV, sex, age at scan and case–control status (Table 3). In the case group, the area and volume of CC segment V and whole CC FA and RD were associated with FSIQ, and in the control group, ICV was associated with FSIQ. No association was found between CC morphometric variables and MABC‐2 total score.

**Table 3:** Multivariable regression associations between FSIQ and CC segment areas, volumes, average FA and RD.

Model	Cohort	Independent variables	Adjusted coefficient *β* (95% CI)	*P* value
1. CC area and FSIQ	Cases and controls	Case status	**−7.1 (−12.39, −1.76)**	0.01*
		ICV (mm^3^)	**3.3 × 10** ^**−5**^ **(0.8 × 10** ^**−5**^ **, 5.8 × 10** ^**−5**^ **)**	0.009*
		Sex	−4.7 (−10.33, 0.85)	0.09
		Age at scan days	0.004 (−0.008, 0.016)	0.51
		CC segment V area (mm^2^)	**0.14 (0.03, 0.26)**	0.01*
	Controls	ICV (mm^3^)	**4.2 × 10** ^**−5**^ **(0.8 × 10** ^**−5**^ **, 7.7 × 10** ^**−5**^ **)**	0.02*
		Sex	−6.5 (−14.97, 1.96)	0.13
		Age at scan days	0.01 (−0.009, 0.035)	0.23
	Cases	ICV (mm^3^)	2.6 × 10^−5^ (−1.0 × 10^−5^, 6.2 × 10^−5^)	0.16
		Sex	−2.7 (−11.19, 5.81)	0.31
		Age at scan days	−0.002 (−0.02, 0.014)	0.16
		CC segment V area (mm^2^)	**0.18 (0.004, 0.35)**	**0.04** *
2. CC volume and FSIQ	Cases and controls	Case status	**−7.0 (−12.42, −1.66)**	**0.01** *
		ICV mm^3^	**3.3 × 10** ^**−5**^ **(0.9 × 10** ^**−5**^ **, 5.9 × 10** ^**−5**^ **)**	**0.009** *
		Sex	−4.8 (−10.43, 0.85)	0.09
		Age at scan days	0.004 (−0.008, 0.016)	0.54
		CC segment V volume (mm^3^)	**0.03 (0.003, 0.05)**	**0.03** *
	Controls	ICV mm^3^	**4.2 × 10** ^**−5**^ **(0.8 × 10** ^**−5**^ **, 7.7 × 10** ^**−5**^ **)**	**0.02** *
		Sex	−6.5 (−14.9, 1.96)	0.13
		Age at scan days	0.013 (−0.009, 0.035)	0.23
	Cases	ICV mm^3^	2.4 × 10^−5^ (−1.3 × 10^−5^, 6.1 × 10^−5^)	0.19
		Sex	−2.3 (−10.88. 6.14)	0.58
		Age at scan days	−0.003 (−0.019, 0.013)	0.73
		CC segment V volume (mm^3^)	**0.04 (0.001, 0.079)**	**0.045** *
3. CC FA and FSIQ	Cases and controls	Case status	**−7.1 (−13.19, −1.05)**	**0.02** *
		ICV (mm^3^)	**4.3 × 10** ^**−5**^ **(1.9 × 10** ^**−5**^ **, 6.8 × 10** ^**−5**^ **)**	**<0.001** **
		Sex	−3.4 (−9.83, 3.09)	0.30
		Age at scan days	0.005 (−0.01, 0.02)	0.5
		Whole CC FA	**10.3 (0.89, 19.89)**	**0.03** *
	Controls	ICV (mm^3^)	**4.2 × 10** ^**−5**^ **(0.7 × 10** ^**−5**^ **, 7.7 × 10** ^**−5**^ **)**	**0.02** *
		Sex	−6.1 (−14.68, 2.47)	0.16
		Age at scan days	0.014 (−0.008, 0.036)	0.20
	Cases	ICV (mm^3^)	3.0 × 10^−5^ (−0.8 × 10^−5^, 6.8 × 10^−5^)	0.11
		Sex	3.6 (−7.55, 14.77)	0.51
		Age at scan days	−0.008 (−0.031, 0.015)	0.48
		Whole CC FA	**13.8 (0.6, 27.1)**	**0.04** *
4. CC RD and FSIQ	Cases and controls	Case status	**−7.5 (−13.38, −1.57)**	**0.01** *
		ICV (mm^3^)	**3.8 × 10** ^**−5**^ **(1.3 × 10** ^**−5**^ **, 6.3 × 10** ^**−5**^ **)**	**0.004** **
		Sex	−3.0 (−9.44, 3.38)	0.34
		Age at scan days	0.007 (−0.009, 0.02)	0.39
		Whole CC RD	**−9.6 (−17.29, −1.92)**	**0.01** *
	Controls	ICV (mm^3^)	**4.2 × 10** ^**−5**^ **(0.7 × 10** ^**−5**^ **, 7.7 × 10** ^**−5**^ **)**	**0.02** *
		Sex	−6.1 (−14.68, 2.47)	0.16
		Age at scan days	0.014 (−0.008, 0.036)	0.20
	Cases	ICV (mm^3^)	2.35 × 10^−5^ (−1.7 × 10^−5^, 6.5 × 10^−5^)	0.25
		Sex	3.0 (−7.91, 14.00)	0.57
		Age at scan days	−0.006 (−0.029, 0.018)	0.61
		Whole CC RD	**−11.3 (−22.22, −0.42)**	**0.04** *

Regression models are adjusted for intracranial volume, sex and age at the scan. Significant predictors are in bold. CC, corpus callosum; FA, fractional anisotropy; FSIQ, full‐scale IQ; ICV, intracranial volume; RD, radial diffusivity.

* *P* < 0.05.

** *P* < 0.005.

#### Tract‐based spatial statistics

No difference in dependence of either FSIQ or MABC‐2 total score on either FA or RD in any segments of the CC between cases and controls was observed.

## Discussion

In a cohort of children aged 6–8 years treated with TH for neonatal HIE without CP, smaller posterior CC area, volume and altered CC diffusion metrics were associated with lower cognitive scores in childhood (early school age) compared to healthy matched controls. Reduced CC growth trajectory from birth to childhood was observed in cases at risk of cognitive impairment (FSIQ ≤85). As reduced CC area, volume and altered diffusion metrics in childhood were associated with combined although mild BGT/WM injury on neonatal MRI in the absence of overt CC injury, these differences observed in the CC are likely linked to Wallerian degeneration^39^ from regions of the brain connecting through the CC. No such associations were observed with motor scores. Together, these findings suggest CC development is impacted by the secondary effects of HIE‐associated brain injury, affecting cognitive outcomes in this population.

In childhood, the CC plays a fundamental role in facilitating cognitive and motor functioning by integrating information across the hemispheres.^40^ Higher CC volumes are linked to higher intelligence,^41^ motor skills^42^ and better problem‐solving abilities in typical childhood development,^43^ and lower CC volumes have been linked to poorer clinical outcomes in neurodevelopmental disorders such as CP.^44^ In the present study, children cooled for HIE at birth had reduced CC area and volume by early school age, even in the absence of CP. The reduction in CC area and volume, due largely to an independent reduction in segment II size, persisted after controlling for age, sex and ICV. These findings suggest long‐term tissue damage associated with HIE in the CC can occur despite cooling with TH and may reflect impaired interhemispheric communication in this population. This is supported by previous work in this cohort that identified altered WM connections via the CC and demonstrated associations between these connections and functional outcomes.^4^

Consistent with our findings in a smaller sample of the current cohort, cases obtained lower scores on tests of motor and cognitive function at school age.^5^, ^45^ Cognitive performance was independently associated with CC segment V area and volume in cases. The posterior CC segment (splenium) contains reciprocal connections between parietal, temporal and occipital areas that support cognitive functions including perception and language,^21^ potentially explaining the association with FSIQ. While no studies have examined this relationship with school‐age cognition, in non‐cooled infants, splenium size has been associated with MABC‐2 scores at 9–10 years.^46^ While we found no such association in our study (likely due to milder impairments associated with TH treatment and exclusion of CP), the MABC‐2 contains an array of cognitive components such as attention and visual perception, which may partly explain the discrepancy between our findings and the aforementioned study.

The microstructural analyses echoed the findings of morphological differences in the CC between cases and controls. Comparison of diffusion metrics demonstrated reduced FA and increased RD spanning segments II–V in cases, implicating a role of de‐ or dysmyelination for which RD is sensitive.^47^ Microstructural abnormalities related to CC myelination have been linked to neurodevelopmental impairments in preterm infants and other developmental disorders.^40^ Microstructural CC alteration may affect whole‐brain structural connectivity, as children cooled for neonatal HIE also demonstrate altered network connectivity and reduced FA in several WM tracts.^4^

In the current study, regression analyses revealed that FA and RD across the entire CC were associated with reduced cognitive performance in cases but not controls, suggesting fewer interhemispheric fibres or reduced axonal myelination contribute to impaired cognition. This is consistent with previous findings in this cohort demonstrating case–control differences in the association between structural connectivity and FSIQ in a network comprising exclusively interhemispheric WM connections.^4^ Demyelination in the CC in other diseases such as multiple sclerosis has also been linked to cognitive performance associated with processing speed and flexibility, attention, working memory and calculation,^48^ thought to reflect impaired signalling integration across brain regions to perform cognitive tasks.^49^ These findings suggest reduced CC signalling may be associated with impaired cognitive function in children cooled for neonatal HIE.

In this cohort, mild neonatal brain injury linked to combined BGT/WM damage was associated with reduced CC volume and increased RD in childhood. While CC thinning following global hypoxic–ischaemic brain injury has been described,^46^ an association between combined mild neonatal BGT and WM injury and CC growth has not previously been identified. In cooled infants with HIE, CC lesions (restricted diffusion on Apparent Diffusion Coefficient maps) on neonatal MRI have primarily been seen with severe BGT lesions or a ‘near‐total’ pattern of brain injury,^6^ linked to glutamate neurotoxicity and subsequent acute cytotoxic oedema.^50^, ^51^, ^52^ While we were unable to measure CC anisotropy on neonatal scans, qualitative assessment determined CC appearance was normal in all cases. This is possibly due to later imaging in our cohort compared to other studies (postnatal age 8 vs. 5 days).^6^ However, the absence of neonatal CC abnormalities suggests the altered diffusion found at 6–8 years emerges later in childhood. This is supported by children in this cohort with cognitive impairment demonstrating inhibited growth of the whole CC including the anterior, mid and posterior third of CC by childhood.

Finally, we also found evidence of sexual dimorphism. In male cases, there was an interaction between case status and sex on the whole CC volume. In a longitudinal study examining volumetric CC trajectories from infancy to early adulthood, ratios of total CC and genu, posterior midbody and splenium volumes to the whole brain were lower in males than females during childhood.^49^ However, in this study, male cases demonstrated lower CC volume to ICV ratios compared to their matched male control peers. These findings therefore suggest males may be differentially susceptible to CC damage following hypoxic–ischaemic injury, warranting further investigation in this population.

### Limitations

Our relatively small sample size may have precluded detecting an association between motor scores and CC metrics. Further, due to the neonatal acquisition sequences used, we were unable to compute the trajectory of CC volume and microstructure from neonate to childhood, which would have provided a more holistic picture of CC development in this population. Finally, we excluded children with CP from our cohort, limiting the range of data. However, we aimed to investigate changes related to functional outcomes in children without overt physical disabilities in mainstream schooling who may not be identified by current follow‐up protocols following TH for HIE. Given 85% of survivors cooled for HIE do not have CP,^53^ these data should be generalisable to the majority of cooled children.

## Conclusions

Smaller posterior CC area, volume and altered diffusion metrics observed in cases were associated with combined BGT/WM injury on neonatal MRI and lower cognitive scores in childhood. These results help provide a link between lesions on neonatal MRI and cognitive outcome at childhood following neonatal HIE through impaired corpus callosal development, which may support further research into predicting outcome and enabling earlier intervention in this population. Although TH has been successful in reducing deep GM injury following HIE, there are still WM developmental alterations. Therefore, therapeutic interventions should focus on WM protection and development. Ongoing assessments of children's abilities are needed in those without CP attending mainstream school and greater additional support may be required before school age for children without CP following HIE at birth.

## Author Contributions

MT, FMC, JCWB and EC were responsible for the conception and design of the study. HB, APCS, GG, SJ, JCWB and EC were responsible for the acquisition and analysis of data. All authors contributed to writing and editing the manuscript and figures.

## Conflicts of Interest

None declared.
